# Supplementary material for: Multi‐dataset identification of innovative feature genes and molecular mechanisms in keratoconus
Source: J Cell Mol Med. 2024 Sep 19;28(18):e70079. doi: 10.1111/jcmm.70079 (PMC11412914; doi:10.1111/jcmm.70079)
Supplement: Supplementary file 2 — Material S2. [file JCMM-28-e70079-s002.zip › 7.GSEA analysis/6.SNORA74B/KEGG_ADHERENS_JUNCTION.html]

Details for gene set KEGG\_ADHERENS\_JUNCTION[GSEA]

|  || Dataset | SNORA74B.group.cls#h\_versus\_l |
| Phenotype | group.cls#h\_versus\_l |
| Upregulated in class | l |
| GeneSet | KEGG\_ADHERENS\_JUNCTION |
| Enrichment Score (ES) | -0.4178371 |
| Normalized Enrichment Score (NES) | -1.621432 |
| Nominal p-value | 0.037383176 |
| FDR q-value | 0.29322284 |
| FWER p-Value | 0.576 |
Table: GSEA Results Summary

  

Fig 1: Enrichment plot: KEGG\_ADHERENS\_JUNCTION      
 Profile of the Running ES Score & Positions of GeneSet Members on the Rank Ordered List

  

| SYMBOL | TITLE | RANK IN GENE LIST | RANK METRIC SCORE | RUNNING ES | CORE ENRICHMENT || 1 | RAC3 | na | 1795 | 0.390 | -0.0034 | No |
| 2 | IGF1R | na | 6399 | 0.180 | -0.1139 | No |
| 3 | BAIAP2 | na | 7054 | 0.161 | -0.1128 | No |
| 4 | FARP2 | na | 8203 | 0.131 | -0.1296 | No |
| 5 | TGFBR2 | na | 9513 | 0.102 | -0.1546 | No |
| 6 | CSNK2A1 | na | 9893 | 0.095 | -0.1538 | No |
| 7 | ACP1 | na | 10508 | 0.083 | -0.1612 | No |
| 8 | CREBBP | na | 10525 | 0.083 | -0.1514 | No |
| 9 | TJP1 | na | 11082 | 0.073 | -0.1583 | No |
| 10 | CTNND1 | na | 11150 | 0.072 | -0.1512 | No |
| 11 | ACTB | na | 11421 | 0.068 | -0.1505 | No |
| 12 | PTPRF | na | 12367 | 0.055 | -0.1710 | No |
| 13 | ACTN4 | na | 12377 | 0.055 | -0.1644 | No |
| 14 | TCF7L2 | na | 12663 | 0.051 | -0.1663 | No |
| 15 | ERBB2 | na | 12767 | 0.050 | -0.1631 | No |
| 16 | SMAD4 | na | 13214 | 0.045 | -0.1704 | No |
| 17 | SMAD2 | na | 13222 | 0.045 | -0.1651 | No |
| 18 | PTPRJ | na | 13952 | 0.036 | -0.1816 | No |
| 19 | IQGAP1 | na | 14103 | 0.035 | -0.1817 | No |
| 20 | LMO7 | na | 14245 | 0.033 | -0.1816 | No |
| 21 | INSR | na | 14317 | 0.032 | -0.1797 | No |
| 22 | SNAI1 | na | 15099 | 0.024 | -0.1993 | No |
| 23 | CDH1 | na | 15137 | 0.023 | -0.1975 | No |
| 24 | PARD3 | na | 15192 | 0.023 | -0.1962 | No |
| 25 | SORBS1 | na | 15813 | 0.016 | -0.2121 | No |
| 26 | CTNNA1 | na | 16086 | 0.014 | -0.2182 | No |
| 27 | PTPN1 | na | 16124 | 0.013 | -0.2176 | No |
| 28 | SMAD3 | na | 16318 | 0.011 | -0.2218 | No |
| 29 | WAS | na | 16570 | 0.009 | -0.2279 | No |
| 30 | MAPK3 | na | 16753 | 0.007 | -0.2323 | No |
| 31 | WASF2 | na | 17499 | -0.001 | -0.2537 | No |
| 32 | WASL | na | 17732 | -0.003 | -0.2601 | No |
| 33 | CTNNB1 | na | 17783 | -0.003 | -0.2611 | No |
| 34 | ACTG1 | na | 18268 | -0.009 | -0.2739 | No |
| 35 | RHOA | na | 18290 | -0.009 | -0.2734 | No |
| 36 | CDC42 | na | 18770 | -0.015 | -0.2854 | No |
| 37 | CTNNA2 | na | 18831 | -0.015 | -0.2852 | No |
| 38 | RAC2 | na | 18892 | -0.016 | -0.2850 | No |
| 39 | NLK | na | 18915 | -0.016 | -0.2836 | No |
| 40 | EGFR | na | 19596 | -0.024 | -0.3003 | No |
| 41 | RAC1 | na | 19881 | -0.027 | -0.3051 | No |
| 42 | CSNK2B | na | 20420 | -0.033 | -0.3165 | No |
| 43 | MAPK1 | na | 20606 | -0.035 | -0.3175 | No |
| 44 | CSNK2A2 | na | 20870 | -0.039 | -0.3203 | No |
| 45 | WASF1 | na | 22378 | -0.057 | -0.3568 | No |
| 46 | VCL | na | 22408 | -0.057 | -0.3505 | No |
| 47 | YES1 | na | 23375 | -0.071 | -0.3696 | No |
| 48 | MAP3K7 | na | 23787 | -0.078 | -0.3718 | No |
| 49 | PTPN6 | na | 24209 | -0.084 | -0.3736 | No |
| 50 | EP300 | na | 25385 | -0.105 | -0.3944 | No |
| 51 | CTNNA3 | na | 25470 | -0.107 | -0.3836 | No |
| 52 | FER | na | 25701 | -0.111 | -0.3764 | No |
| 53 | TGFBR1 | na | 27137 | -0.142 | -0.4002 | Yes |
| 54 | TCF7L1 | na | 27185 | -0.144 | -0.3837 | Yes |
| 55 | WASF3 | na | 28101 | -0.167 | -0.3893 | Yes |
| 56 | ACTN1 | na | 28221 | -0.171 | -0.3716 | Yes |
| 57 | MET | na | 28809 | -0.189 | -0.3650 | Yes |
| 58 | TCF7 | na | 28810 | -0.189 | -0.3415 | Yes |
| 59 | SRC | na | 30227 | -0.234 | -0.3533 | Yes |
| 60 | ACTN2 | na | 30345 | -0.239 | -0.3270 | Yes |
| 61 | SSX2IP | na | 30663 | -0.251 | -0.3050 | Yes |
| 62 | PTPRM | na | 30762 | -0.256 | -0.2760 | Yes |
| 63 | SNAI2 | na | 31910 | -0.318 | -0.2696 | Yes |
| 64 | PTPRB | na | 32613 | -0.361 | -0.2450 | Yes |
| 65 | ACTN3 | na | 33200 | -0.413 | -0.2106 | Yes |
| 66 | FYN | na | 33540 | -0.454 | -0.1639 | Yes |
| 67 | LEF1 | na | 34351 | -0.633 | -0.1087 | Yes |
| 68 | FGFR1 | na | 34687 | -0.963 | 0.0012 | Yes |
Table: GSEA details [plain text format]

  

Fig 2: KEGG\_ADHERENS\_JUNCTION      
 Blue-Pink O' Gram in the Space of the Analyzed GeneSet

  

Fig 3: KEGG\_ADHERENS\_JUNCTION: Random ES distribution      
 Gene set null distribution of ES for **KEGG\_ADHERENS\_JUNCTION**
